# EGFR and HER3 expression in circulating tumor cells and tumor tissue from non-small cell lung cancer patients

**DOI:** 10.1038/s41598-019-43678-6

**Published:** 2019-05-15

**Authors:** Heather Scharpenseel, Annkathrin Hanssen, Sonja Loges, Malte Mohme, Christian Bernreuther, Sven Peine, Katrin Lamszus, Yvonne Goy, Cordula Petersen, Manfred Westphal, Markus Glatzel, Sabine Riethdorf, Klaus Pantel, Harriet Wikman

**Affiliations:** 1 Department of Tumour Biology, University Medical Centre Hamburg-Eppendorf, Hamburg, Germany; 2 Department of Oncology, Hematology and Bone Marrow Transplantation with section Pneumology, University Medical Centre Hamburg-Eppendorf, Hamburg, Germany; 3 Department of Neurosurgery, University Medical Centre Hamburg-Eppendorf, Hamburg, Germany; 4 Department of Neuropathology, University Medical Center Hamburg-Eppendorf, Hamburg, Germany; 5 Institute for Transfusion Medicine, University Medical Center Hamburg-Eppendorf, Hamburg, Germany; 6 Department of Radiotherapy, University Medical Centre Hamburg-Eppendorf, Hamburg, Germany

**Keywords:** Isolation, separation and purification, Non-small-cell lung cancer, Non-small-cell lung cancer

## Abstract

Although clinically relevant, the detection rates of EpCAM positive CTCs in non-small cell lung cancer (NSCLC) are surprisingly low. To find new clinically informative markers for CTC detection in NSCLC, the expression of EGFR and HER3 was first analyzed in NSCLC tissue (n = 148). A positive EGFR and HER3 staining was observed in 52.3% and 82.7% of the primary tumors, and in 62.7% and 91.2% of brain metastases, respectively. Only 3.0% of the brain metastases samples were negative for both HER3 and EGFR proteins, indicating that the majority of metastases express these ERBB proteins, which were therefore chosen for CTC enrichment using magnetic cell-separation. Enrichment based on either EGFR or HER3 detected CTCs in 37.8% of the patients, while the combination of EGFR/HER3 enrichment with the EpCAM-based CellSearch technique detected a significantly higher number of 66.7% CTC-positive patients (Cohen’s kappa = −0.280) which underlines the existence of different CTC subpopulations in NSCLC. The malignant origin of keratin-positive/CD45-negative CTC clusters and single CTCs detected after EGFR/HER3 based enrichment was documented by the detection of NSCLC-associated mutations. In conclusion, EGFR and HER3 expression in metastasized NSCLC patients have considerable value for CTC isolation plus multiple markers can provide a novel liquid biopsy approach.

## Introduction

The field of precision medicine is evolving rapidly. For non-small cell lung cancer (NSCLC), which is the most common cause of cancer-related death world wide^1^, personalized strategies using targeted therapies are already of great benefit for a subset of patients harboring specific somatic aberrations. However, the occurrence of secondary mutations that drive resistance to targeted therapies, poses a big challenge in clinical oncology^2^. In addition to the prognostic power of circulating tumor cell (CTC) analysis, CTCs could also serve as a valuable real-time tool for monitoring therapy and disease recurrence in cancer patients^3,4^. Due to the non-invasiveness of the procedure, CTC analysis allows serial monitoring of patients and can provide information e.g. on the mutation burden from different metastatic sites^5^ or the occurrence of resistance mutations^6^. CTC detection is still challenging, especially for NSCLC patients, and numerous different CTC detection systems have been proposed^7–9^. With the most commonly used EpCAM based CTC detection systems the CTC detection rate in NSCLC patients is surprisingly low, especially when considering the aggressiveness of the disease^10^. The reported low CTC numbers might be caused by an epithelial-mesenchymal transition (EMT) of the cancer cells reaching the blood stream, which could entail downregulation of the epithelial marker EpCAM^11–13^. Furthermore, the pronounced tumor heterogeneity, a characteristic of NSCLC tumor tissue, creates an additional big challenge in liquid biopsy research^3,14^. Making use of multiple surface molecules as antigens that are specific for the tumor tissue could improve CTC isolation^15^.

The Epidermal Growth Factor Receptor (ERBB)-family of receptor tyrosine kinases is of great biological and clinical importance in NSCLC. In tumor cells the ERBB proteins regulate proliferation, differentiation and apoptosis^16^. Approximately 10–15% of Caucasian lung adenocarcinoma patients and up to 40% of patients of Asian origin carry an activating mutation of the EGF-receptor (EGFR)^17^. These patients can efficiently be treated with different small molecule inhibitors (tyrosine kinase inhibitors) targeting EGFR^18^. However, most patients will eventually develop resistance against these therapies. Upregulation of HER3 expression has been found to be one of the mechanisms that drive EGFR resistance^19,20^.

Here, we first screened the EGFR and HER3 expression in primary and metastatic NSCLC tumor tissue. Based on the results, we developed a CTC detection technique using these two markers and show that in combination with EpCAM we could capture a larger fraction of CTCs in NSCLC patients.

## Materials & Methods

### Ethical statement

The institutional review board of the ethical commission of the Hamburger Ärztekammer (PV3779) approved this study. Patients were enrolled between September 2015 and May 2016 and informed consent was obtained from all participants. All research was performed in accordance with relevant guidelines and regulations.

### Patients

Tissue microarrays (TMA) with 55 surgical tissue specimens from histologically proven NSCLC primary tumors, 17 matched lymph node metastases and 76 non-matched lung cancer brain metastases were used for immunohistochemistry (IHC) staining of EGFR and HER3 proteins. For each patient, tissue was analyzed in duplicates with a core size of 1.0 mm. The primary tumor TMA has been described in detail before^21^. 40.0% of the samples were adenocarcinomas, 38.2% squamous cell carcinoma, and 21.8% of other subtypes. 58.2% were males and 41.8% females. 31.5% were diagnosed with stage I disease, 29.6 with stage II and 38.9 stage III. 98% of patients were current or ex-smokers and only one patient was a non-smoker (5 patients had unknown smoking status). In the brain metastases TMA (non-matched to the primary tumors), 75.4% of the samples were adenocarcinomas, 13.8% squamous cell carcinoma, and 10.8% other or unclear subtype. The average age of brain metastases diagnosis was 60.3 years (range: 32–78) and 56.6% were males and 43.4% females.

Blood was drawn from a cohort of 45 advanced stage (Stage III or IV) NSCLC patients at first diagnoses or at disease progression for CTC isolation. Patients were enrolled from the Department of Oncology, Hematology and Bone Marrow Transplantation with section Pneumology, the Department of Neurosurgery or from the Department of Radiation Therapy, all part of the University Medical Center Hamburg-Eppendorf (UKE, Germany). Fifteen patients were recruited prior to resection of brain metastases. Of the total cohort, 41 patients were diagnosed with adenocarcinoma (AC), three with squamous cell carcinoma and one with large-cell carcinoma. Six of the AC patients received anti-EGFR therapy. Patient characteristics are shown in Table 1. In addition, 18 healthy donor samples were enrolled from the Department for Transfusion Medicine (UKE). For HER3/EGFR CTC enrichment, 7.5 ml of blood were collected in EDTA tubes (Sarstedt, Nuernbrecht, Germany) whereas CellSave tubes (Janssen Diagnostics, Raritan, New Jersey) were used for CellSearch CTC analyses. Blood samples were stored at room temperature. The EDTA blood was processed within 6 hours and the CellSave blood within 24 h.

**Table 1:** Clinical characteristics of NSCLC patients from the MACS cohort. n.a.: data not available.

		*n*	%
*Histology*	AC	41	91.1
	SCC	3	6.7
	other	1	2.2
*Gender*	Female	20	44.4
	Male	25	55.6
*Age*	Mean (range)	63 (41–85)	
*UICC stage*	III	5	11.1
	IV	40	88.9
*Metastatic state*	Oligo-met.	14	31.1
	Multiple met.	27	60.0
	M0	4	8.89
*Mutation status*	EGFR mut.	7	15.6
	Wild type	30	66.7
	n.a.	8	17.8

### Immunohistochemistry staining of lung primary tumor and brain metastases tissue for EGFR and HER3 protein expression

TMA slides were first baked for 1 hour at 60 °C, followed by a de-paraffinization in xylol and a decreasing ethanol series for rehydration purpose. Tissue was treated with either proteinase K (DAKO/Agilent; Santa Clara, California; EGFR) or boiled in 1 mM EDTA (HER3) for antigen unmasking. Peroxidase blocking solution or 5% goat serum in 1xTBST were used for EGFR (1:20; DAKO) or HER3 (1:250; Cell Signaling Technology, Danvers, Massachusetts) immunostainings, respectively and both incubated overnight at 4 °C. For visualization, DAKO Envision Kit or Signal Stain Boost (Cell Signaling Technology) was used. The tissue was counterstained with hemalaun solution for 2 seconds and dehydrated with an increasing ethanol series and a final xylol incubation step. Specimens were covered with Eukitt (ORSAtec, Bobingen, Germany) and a cover glass.

Staining of TMAs was analyzed blinded by two independent scientists (AH and HW). Both signal intensity and signal distribution were multiplied to a final value and grouped according to the final value into negative (0–0.5), intermediate (0.6–2) and strong (≥2) staining. Disconcordant evaluations were analyzed for a second time.

### Establishment of the HER3/EGFR based CTC enrichment method

First, the specificity of different biotinylated EGFR and HER3 antibodies was tested by immunofluorescence staining on cyto-centrifuged MDA-MB-468 (EGFR) or SKBR3 (HER3) cells mixed with healthy donor PBMCs. For EGFR based CTC enrichment, three different biotinylated monoclonal antibodies were tested, including clone 14C8 (Acris Antibodies, Herford, Germany), clone B1D8 and clone Hu1 (both from Novus Biologicals, Littleton, Colorado). For HER3 we tested two different antibodies; the biotinylated human anti-HER3 antibody (clone REA508; Miltenyi Biotec, Bergisch Gladbach, Germany) and the biotinylated human anti-HER-3 antibody (clone 1B4C3; BioLegend, San Diego, California). Cells were fixed with 4% PFA, washed with PBS and permeabilized by 0.1% Tween 20 in PBS. After another washing step the samples were blocked in 5% goat serum in PBS and incubated with the antibodies EGFR (1:50) or HER3 (1:25) for one hour at room temperature (RT). The secondary streptavidin conjugated antibody, *life technologies* (Carlsbad, California), was incubated for 45 min at RT and washed away with PBS. Cells were additionally stained for CD45 (CD45–647, 1:150; Biolegend) and DAPI (1:500; Sigma, St. Louis, Misssouri) for one hour at RT. Based on the intensity and specificity (negative leucocyte staining) of the staining we chose both the tested HER3 antibodies and the EGFR antibody clone B1D8 from Novus Biologicals (NBP2-34553B) for the establishment of a bead enrichment CTC protocol (Supplementary Information 1).

Experiments on recovery rates (Supplementary Information 2) were performed by spiking tumor cells into blood samples from healthy donors (received from the department of transfusion medicine). 50 cells of either the HER3 positive SKBR3 (for HER3 isolation) or 50 EGFR-positive MDA-MB-468 cells (for EGFR isolation) were manually spiked into 4 ml blood. For the HER3-enrichment, two different protocols were tested. The biotinylated HER3-antibody, clone REA508, was incubated at 4 °C for 10 min with PBMCs followed by 15 min incubation with the streptavidin coated magnetic beads. Second, the HER3-antibody clone 1B4C3 was incubated at room temperature for 30 min followed by 60 min magnetic bead incubation. A recovery rate of 67% (range 58–82%; n = 3) was obtained for the HER3-antibody clone REA509 compared to 44% (range 34–52%) when using the HER3-antibody clone 1B4C3. To optimize the protocol for the EGFR- based enrichment (clone B1D8), we compared two concentrations (1:10 and 1:15 dilutions) for the antibody incubation (30 min at RT). Here, the higher concentration showed higher recovery rates; of 64% (range 38–82%; n = 3) compared to 34% (range 26–41%; n = 3). To allow larger studies with multiple sites involved in patient recruitment, ensuring that newly identified CTC isolation techniques are efficient also in blood preservative tubes is of high importance. Therefore, we analyzed spiked blood samples in CellSave tubes (Janssen Diagnostics). Overnight incubation of the samples in CellSave tubes showed a significant reduction on the CTC recovery rates for the EGFR antibody (clone B1D8) with a mean recovery rate of 36% (Supplementary Information 2 C), whereas the HER3 enrichment was not influenced by the blood collection tube. Given that this was a single center study, we therefore collected all patient samples in EDTA tubes.

### CTC Isolation via magnetic cell separation

The PBMC cellular fraction (7.5 ml blood) was enriched using the Leucosep tubes (Greiner Bio-One, Kremsmünster, Austria). PBMCs were divided into two parts and incubated either at 4 °C for 10 minutes (HER3 antibody; Miltenyi) with a dilution of 1:11 in 100 μl cell suspension or for one hour at room temperature (EGFR antibody; 1:10 dilution; Novus Biologicals antibody in 100 μl cell suspension). When working with more than 1 × 10^7^ cells, twice the volume of all indicated volumes were used. The PBMCs were then washed with *MACS running buffer* followed by 15 min incubation with 10 μl streptavidin-coated beads (both Miltenyi) per 1 × 10^7^ cells for the HER3 and 20 μl for 30 min for EGFR. Unspecific binding of magnetic particles was washed away with the *separation buffer* and the cell suspension was applied on a MS Column (Miltenyi). After washing three times with 500 μl *separation buffer* the magnetically labeled cells were finally flushed onto two glass slides, centrifuged and dried overnight. Slides were immunofluorescent stained within 3 days. Cells were fixed with 4% PFA for 10 min, washed with PBS and blocked in 10% AB serum. The antibody cocktail was applied for 45 min at RT. Immunofluorescence staining was performed using pan-Keratin antibody (AE1AE3 eFluor570; 1:80; eBioScience, San Diego, California) as CTC and CD45 (CD45-Alexa647; 1:150; Biolegend) as leukocyte marker. DAPI was applied as nuclear dye (1:500; Sigma). 12 samples were also stained for cMET protein expression (clone: C1D2, Cell Signaling, 1:3000, overnight at 4 °C; secondary antibody: anti-rabbit-alexa 488 (eBioScience, 1:1000, 60 min at RT).

### CellSearch analysis

In parallel, 41 patient samples were analyzed by the CellSearch system (Silicon Biosystems, Bologna, Italy)^22^. The CellSearch System is a semi-automated EpCAM-based CTC enrichment technique. It enriches tumor cells of epithelial origin (EpCAM+) and allows enumeration of CTCs (CD45- and keratins 8, 18, and 19+) in whole blood.

### WGA and quality control

Whole genome amplification (WGA) of single cell CK-positive/CD45-negative CTCs was performed with either the Picoplex™ WGA Single Cell Whole Genome Amplification Kit (Rubicon Genomics, Ann Arbor, Michigan) or the AmpliI Kit (Silicon Biosystems). Single cells were isolated by micromanipulation. 2 μl of Cell lysis buffer (Silicon Biosystems) was added on the cells and the lysate was stored at −80 °C until further usage. WGA was performed according to the manufacturer’s instructions, and DNA products were stored at −80 °C until downstream molecular analysis. The DNA concentration after the WGA was measured by the Qubit™ fluorometer (Thermo Fisher Scientific, Pinneberg, Germany). The quality of the WGA product was assessed by the Ampli1™ QC Kit (Silicon Biosystems). Cells with good quality (three or four PCR bands) were used for the next-generation sequencing (NGS) analyses.

### Statistical analysis

Associations between protein expression (TMA) or CTC status and clinical characteristics were tested by chi-square, two-sided Fisher’s exact test and Kaplan-Meier analysis in line with the logrank test, respectively. The correlation of the Cell Search and the MACS system were assessed using the Cohen’s kappa. P-values below 0.05 were considered significant.

#### Mutation detection

For the targeted detection of somatic mutations in CTCs and tissue we used the iPLEX HS technology in conjunction with the MALDI-TOF based MassARRAY System (Agena Bioscience, San Diego, California). Here we used the Lung Panel covering 70 mutations in five key oncogenes (BRAF, EGFR, ERBB2, KRAS and PIK3CA) implicated in disease progression and therapy response. A mutation signal produced using iPLEX HS chemistry can be reliably detected by the MassARRAY System at about 1% VAF^23^.

## Results and Discussion

The prognostic value of CTC detection with the CellSearch System has already been demonstrated in many previous studies^10^. However, for NSCLC patients in the majority of cases CTCs are undetectable with EpCam based methods, suggesting that the sensitivity of the assay for this purpose might be too low. In this study, we analyzed whether detection sensitivity could be increased by using other surface markers for CTC capture. Our assumption was supported by previous results where we analyzed EpCAM expression in primary and metastatic NSCLC tissues^21^. Furthermore, others and we have previously shown that important tumor markers from the ERRB-family can be specifically upregulated in CTCs and metastatic tissue compared to the primary tumor^24,25^. Therefore, we first screened the expression level of the clinically important target proteins *EGFR* and *HER3* in primary and metastatic tissue from NSCLC patients.

### HER3 and EGFR protein expression

**Figure 1:**
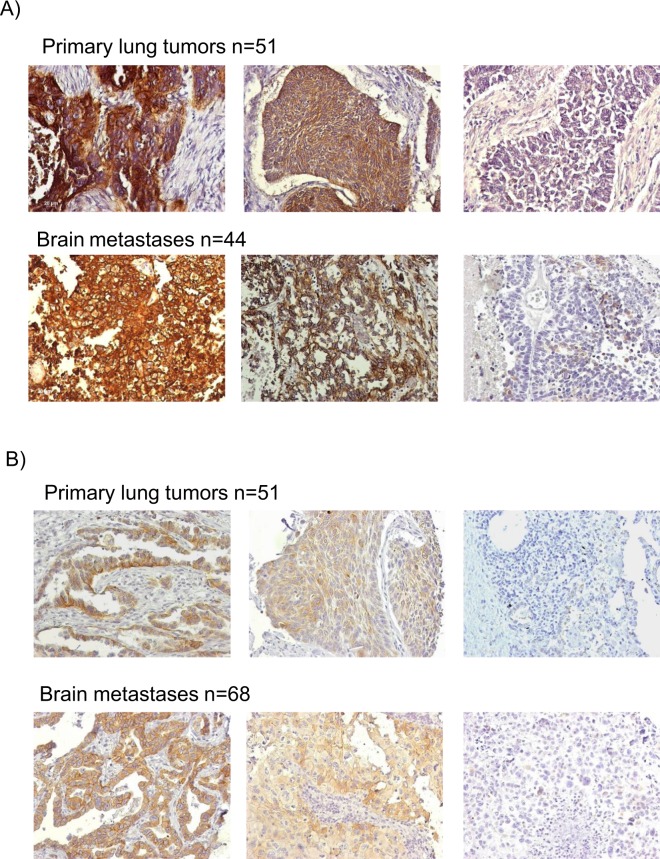
Protein expression and frequency distribution in primary and metastatic lung cancer tissue. EGFR protein immunohistochemistry (**A**) and HER3 protein immunohistochemistry (**B**) was performed on tissue microarray from primary NSCLC tumors and brain metastases. Representative strong (left), intermediate (center) and negative (right) staining are shown in 200x magnification (Zeiss Axiovision).

**Figure 2:**
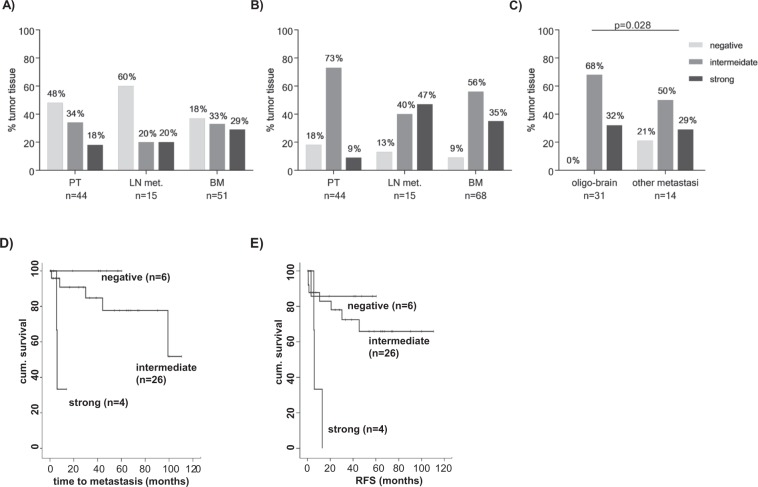
EGFR (**A**) and HER3 (**B**) expression distribution in primary lung, lymph node and brain metastatic tissue and association between HER3 protein expression in primary and clinicopathological parameters (**C**). HER3 is more frequently expressed in brain metastasis of oligo-brain metastatic patients (p = 0.028) compared to patients with other metastatic sites. HER3 expression in primary NSCLC tumors is significantly associated with a decreased time to metastatic progression (p = 0.006; log-rank test) (**D**) and decreased relapse-free survival time (p = 0.013; log-rank test) (**E**).

Several studies have shown that approximately 60% of lung cancer primary tumor tissue display overexpression of *EGFR* at protein level^26–28^. In addition, *HER3* is often expressed in lung tumor tissue^28,29^, whereas less is known about expression in metastatic tissue. Analyzing our TMA, we classified EGFR and HER3 protein expression as either negative, intermediate or strong. Evaluable results were obtained from 44 primary tumors, 15 matched lymph node metastases and 51 non-matched brain metastases. Representative staining are shown in Fig. 1A. Evaluable results for HER3 were obtained from 44 primary tumors, 15 lymph node metastases, and 68 brain metastases samples (Fig. 1B). A positive (intermediate or strong) staining for EGFR was observed in 52.3% of primary tumors, 40.0% of the lymph node metastases, and in 62.7% of brain metastases (Fig. 2A). The proportion of tumors with strong EGFR expression increased from 18.2% in primary tumors to 29.4% in brain metastases, indicating an upregulation of EGFR in metastatic tissue. Whether the higher expression levels in the brain metastases are caused by a *de novo* expression cannot be assessed as non-matched samples were analyzed. No association between EGFR expression in primary tumors and clinical status was found (data not shown). In brain metastases, a significant inverse correlation between the size of the patients’ primary tumors and EGFR expression was found (p = 0.009). EGFR was also linked with a more frequently detection in the brain metastases of squamous cell carcinomas (p = 0.001). 82.7% of primary tumors, 86.6% of lymph nodes and 91.2% of brain metastases were positive for HER3 expression (Fig. 2B). Strong HER3 expression increased from 9.1% in primary tumors to 35.3% in brain metastases. HER3 expression in brain metastases was significantly associated with an oligo-metastatic brain disease state, i.e. brain as the single metastatic site (p = 0.028; n = 31) (Fig. 2C). Survival analyses revealed a significant association of HER3 expression in primary lung tumors with a shorter time to metastatic progression (p = 0.006; n = 36) (Fig. 2D) and a shorter time of relapse-free survival (p = 0.013; n = 36) (Fig. 2E). No further associations between HER3 protein expression and clinical patient characteristics or outcome were found (data not shown). When both EGFR and HER3 staining results were combined, only three brain metastases samples (3.0%) were negative for both HER3 and EGFR proteins, indicating that the large majority of metastases express ERBB proteins. In contrast, we previously showed that 20.9% of brain metastases were negative for EpCAM expression, thus supporting our approach to include additional surface markers for CTC isolation in NSCLC^21^. Moreover, we previously showed that elevated amounts of HER3 mRNA transcripts could be detected in the blood of metastatic NSCLC patients^21^. Based on these results, the here described magnetic CTC enrichment technique was established using biotinylated antibodies to isolate CTCs that express these therapeutically relevant proteins.

### CTC Detection by magnetic cell separation

**Figure 3:**
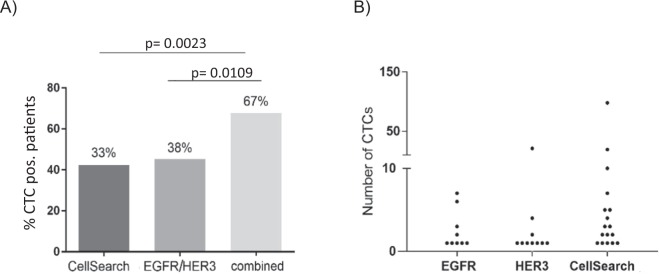
CTC positivity rates in NSCLC patients (n = 45). (**A**) Significantly higher detection rates when combining the methods in comparison to either single use CellSearch (p = 0.0023) or magnetic cell separation with EGFR/HER3 (p = 0.0109). (**B**) CTC enumeration detected by CellSearch and MACS.

**Figure 4:**
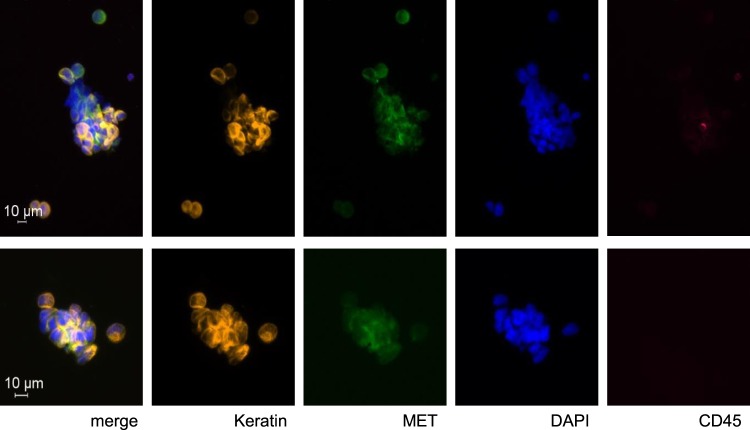
Representative images of two Circulating Tumor Cell cluster (DAPI^+^/CD45^−^/CK^+^) isolated via magnetic cell separation with HER3-protein antibody in one NSCLC patient. Heterogeneous MET expression was found within the cluster as well as CD45-positive leukocytes.

For detection of different CTCs subpopulations, a two-step detection and identification protocol was established. In the first step, the CTCs were enriched out of the vast amount of PBMCs via a positive selection based on HER3 and/or EGFR protein expression. This was followed by a second step of immunofluorescence staining (pan-keratin and CD45) of the enriched cell fraction. A sample was defined as CTC positive if at least one CTC (keratin positive/CD45 negative) was detected. Blood samples from 45 metastatic NSCLC patients and 18 healthy donors (HD) were analyzed with this MACS based technique. The EGFR/HER3-based method was negative for all HD samples and positive in 37.8% of the patients (mean: 3 CTCs; range: 1–21 CTCs) (Fig. 3). In one patient 10 large CTC clusters and 21 single CTCs could be observed (Fig. 4). Furthermore, the EGFR/HER3-technique was positive in 44% (n = 24) of patients suffering from brain metastases, which is in line with our data obtained from tissue staining thus supporting the specific involvement of ERBB pathway members in brain metastasis formation. Noteworthy, we have recently shown that patients with brain metastases are less often CTC positive when analyzed by the CellSearch method^30^. This new method shows a higher suitability when analyzing patients with brain metastases.

In addition to HER3 overexpression, also MET amplification is a known mechanism of acquired resistance against EGFR tyrosine kinase inhibitors^31^. We therefore analyzed MET expression of keratin positive CTCs in twelve patients. In five of these patients keratin positive CTCs were detected. In three patients the detected keratin-positive CTCs (1–2 CTCs) were negative for MET whereas in the fourth patient the one CTC was positive for both MET and keratins. The fifth positive case with many single CTCs and clusters had a heterogeneous MET expression in both single CTCs, as well as in the clusters (Fig. 4), indicating that MET can be neither used for CTC isolation nor as identification marker of CTC.

In 39 parallel samples CellSearch analyses were performed. In 33.4% of the patients ≥1 CTCs/ 7.5 ml blood was detected and in 22.0% ≥3 CTCs were detected. 12.8% of the patients with brain metastases had ≥1 CTC/ 7.5 ml blood. There was no significant correlation in CTC positivity with either method or EGFR mutation status. Combining both CTC detection methods (≥1 CTC) revealed CTCs in 66.7% of NSCLC patients. Interestingly, in only four patients CTCs were detected by both methods (significant negative correlation by Cohen’s kappa = −0.280), underlining the vast heterogeneity of CTCs in NSCLC. Further research is needed to specifically characterize these subpopulations in order to draw a conclusion on their role in the disease and their clinical impact on the disease and treatment course. In one AC patient with numerous clusters and single CTCs detected by EGFR/HER3 based enrichment method only two CTCs were found when analyzed with the CellSearch system. Clusters have been described to possess an up to 50-fold increased higher metastatic potential^32,33^ and are of pathophysiological relevance for tumor spread^34^. It remains unclear which genetic and biological variation determines a patients’ prognosis and the best possible treatment. The number of single CTCs and clusters isolated remain small though and further research is needed to characterize NSCLC subpopulations.

### Mutation detection in CTCs

**Figure 5:**
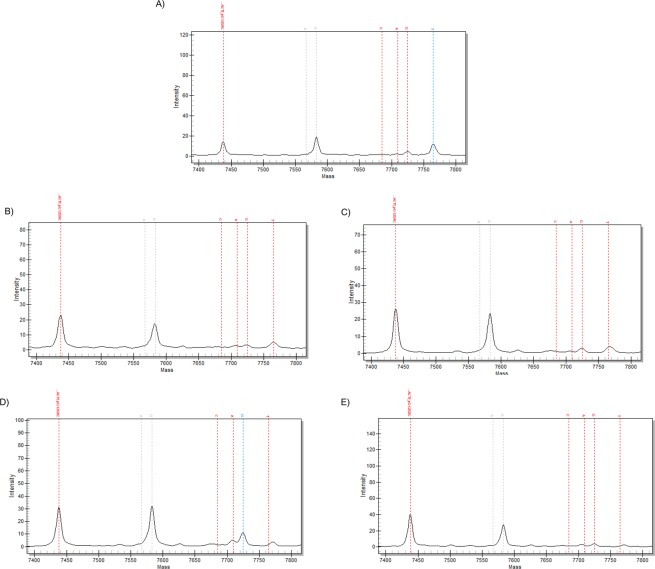
Molecular analysis of primary lung tumor tissue (PT), corresponding circulating tumor cell cluster and three single CTCs. MassARRAY system (iPLEx Lung) shows heterozygous mutation for KRAS G12S in PT. (**A**) Same mutation is seen in the CTC cluster (**B**) and in one single CTC (**C**), whereas two single CTCs (**D**,**E**) show no mutation for KRAS.

In order to prove the tumorigenic origin of CTCs detected by our new approach, we analyzed CTCs and the corresponding primary tumor tissue from the patient where numerous CTCs and clusters were isolated (Fig. 5). The success of the WGA was proven by a multiplex PCR. Based on the WGA results three single CTCs and one cluster as well as the primary tumor tissue was analyzed for 70 hot spot mutations in five genes using the MassARRAY system^23^. The primary tumor tissue showed a heterozygous mutation for KRAS G12S. The same mutation could be detected in the CTC cluster and in one of the CTCs, whereas two CTCs had a low amplification yield and no mutations could be detected. The results above prove that this new method allows further genomic analyzes of CTCs and precludes description of false-positive epithelial cells.

## Conclusion

In conclusion, this study demonstrates the large heterogeneity of NSCLC CTCs and thus the benefit of the use of multiple markers for both CTC enrichment and detection. By combining EpCAM enrichment with EGFR and HER3 based enrichment, CTCs can be detected in a large proportion of patients, including brain metastatic patients who were previously found to have only very few EpCAM positive CTCs^21^. We further show that the isolated CTC are suitable for downstream molecular characterization, which may provide important information in CTC biology relevant to cancer therapy. However, for the feasibility of implementation multi-centered studies further tests on using blood collection tubes with preservatives must be accomplished.

## Supplementary information

Supplementary figure

## Data Availability

The datasets generated and analyzed during the current study are available from the corresponding author on reasonable request.
