# Supplementary material for: EGFR and HER3 expression in circulating tumor cells and tumor tissue from non-small cell lung cancer patients
Source: Sci Rep. 2019 May 15;9:7406. doi: 10.1038/s41598-019-43678-6 (PMC6520391; doi:10.1038/s41598-019-43678-6)
Supplement: Supplementary file 1 — Supplementary figure [file 41598_2019_43678_MOESM1_ESM.pdf]

Supplementary information

EGFR and HER3 expression in circulating tumor cells and tumor tissue from non-small cell lung cancer patients

Heather Scharpenseel[1#], Annkathrin Hanssen[1#], Sonja Loges[2], Malte Mohme[3], Christian Bernreuther[4], Sven Peine[5], Katrin Lamszus[3], Yvonne Goy[6], Cordula Petersen[6], Manfred Westphal[3], Markus Glatzel[4], Sabine Riethdorf[1], Klaus Pantel[1], Harriet Wikman[1†]

[1] Department of Tumour Biology, University Medical Centre Hamburg-Eppendorf, Hamburg, Germany.
[2] Department of Oncology, Hematology and Bone Marrow Transplantation with section Pneumology, University Medical Centre Hamburg-Eppendorf, Hamburg, Germany.
[3] Department of Neurosurgery, University Medical Centre Hamburg-Eppendorf, Hamburg, Germany.
[4] Department of Neuropathology, University Medical Center Hamburg-Eppendorf, Hamburg, Germany.
[5] Institute for Transfusion Medicine, University Medical Center Hamburg-Eppendorf, Hamburg, Germany.
[6] Department of Radiotherapy, University Medical Centre Hamburg-Eppendorf, Hamburg, Germany.

1

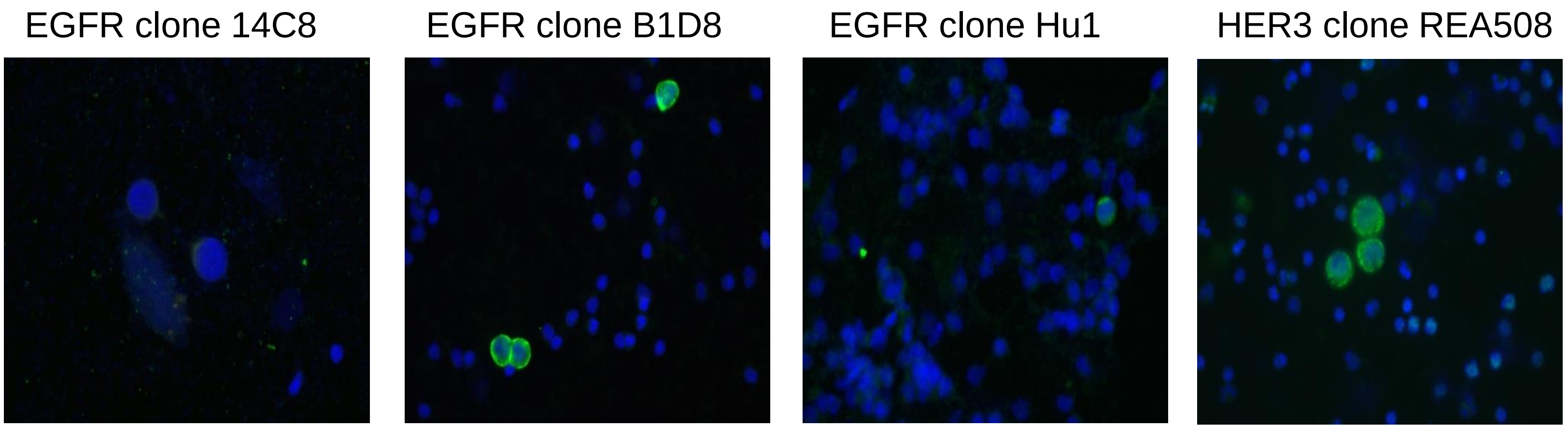

2

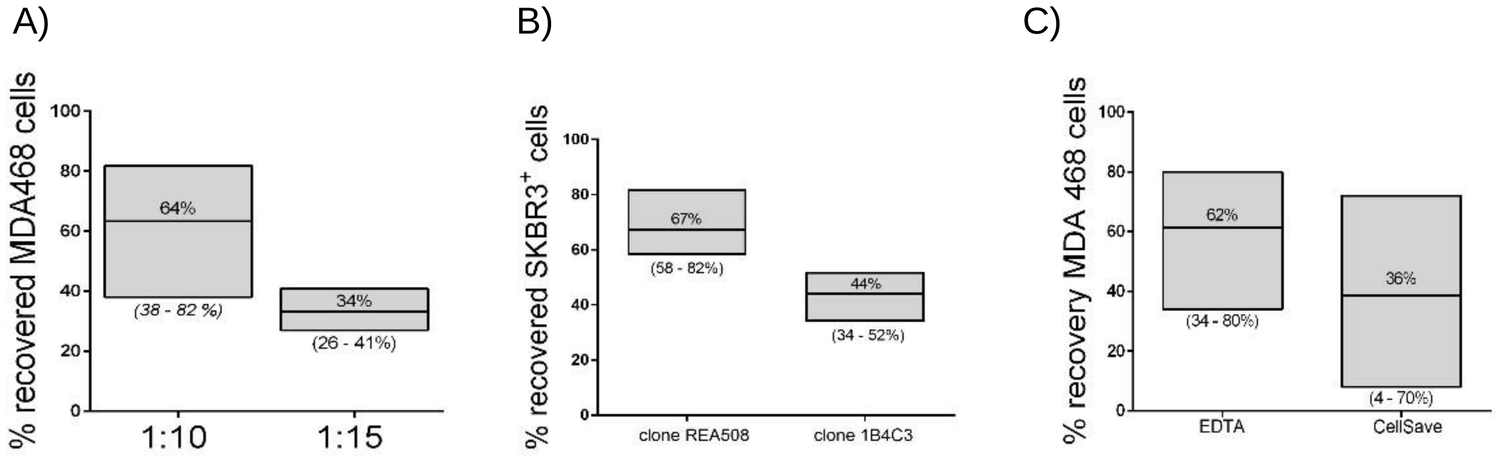

1 To test the specifity of different biotinylated EGFR and HER3 antibodies, we performed immunofluorescence staining on MDA-MB-468 (for EGFR) and SKBR3 (HER3) cells in blood**.** **2** Establishment of a HER3/EGFR based CTC enrichment method performing spiking experiments. Several protocols were tested for higher recovery rates. The EGFR-antibody, clone B1D8, was tested in both 1:10 and 1:15 concentrations on EGFR-positive MDA-MB-468 cells (n=3). (C) Two HER3-antibodies, clone REA508 and clone 1B4C3, were simultaneously tested on the protocol (n=3) (B). Using EGFR antibody, clone B1D8, on MDA-MB-468 cells, EDTA tubes were tested in comparison to CellSave tubes (n=3) (C).
